# A Multi-Stage Deep-Learning-Based Vehicle and License Plate Recognition System with Real-Time Edge Inference

**DOI:** 10.3390/s23042120

**Published:** 2023-02-13

**Authors:** Adel Ammar, Anis Koubaa, Wadii Boulila, Bilel Benjdira, Yasser Alhabashi

**Affiliations:** Robotics and Internet-of-Things Lab, Prince Sultan University, Riyadh 11586, Saudi Arabia

**Keywords:** vehicle identification, license plate recognition, tracking, deep learning, computer vision, video analytics

## Abstract

Video streaming-based real-time vehicle identification and license plate recognition systems are challenging to design and deploy in terms of real-time processing on edge, dealing with low image resolution, high noise, and identification. This paper addresses these issues by introducing a novel multi-stage, real-time, deep learning-based vehicle identification and license plate recognition system. The system is based on a set of algorithms that efficiently integrate two object detectors, an image classifier, and a multi-object tracker to recognize car models and license plates. The information redundancy of Saudi license plates’ Arabic and English characters is leveraged to boost the license plate recognition accuracy while satisfying real-time inference performance. The system optimally achieves real-time performance on edge GPU devices and maximizes models’ accuracy by taking advantage of the temporally redundant information of the video stream’s frames. The edge device sends a notification of the detected vehicle and its license plate only once to the cloud after completing the processing. The system was experimentally evaluated on vehicles and license plates in real-world unconstrained environments at several parking entrance gates. It achieves 17.1 FPS on a Jetson Xavier AGX edge device with no delay. The comparison between the accuracy on the videos and on static images extracted from them shows that the processing of video streams using this proposed system enhances the relative accuracy of the car model and license plate recognition by 13% and 40%, respectively. This research work has won two awards in 2021 and 2022.

## 1. Introduction

With the significant advances in artificial intelligence (AI) in recent years, AI-based surveillance is a growing market worldwide. The global AI surveillance camera market was reported to have reached USD 4 billion in 2022 and is projected to reach USD 13.76 billion by 2027, with a compound annual growth rate (GACR) of 19.9% [1]. Within this market share, the automatic number plate recognition (ANPR) market takes a considerable percentage. The ANPR system market is projected to evolve from USD 3.1 billion in 2022 to USD 4.8 billion in 2027, with a GACR rate of 9.2% between 2022 and 2027 [2]. In other words, the ANPR market is estimated to take one-third of the global market for AI-based surveillance by 2027.

The primary factors propelling the expansion of the ANPR market are many and could be summarized in six factors. The first factor is the high need to deploy ANPR systems in security, smart surveillance, and intelligent traffic management systems. Secondly, the global increase in infrastructure facilities allows the deployment of such systems, especially in developing countries. The third factor is the government’s rising allocation of funds to intelligent transportation systems (ITSs). The fourth factor is the rapidly increasing use of video analytics technologies for vehicle activity monitoring. Fifth is the increasing demand for access control, smart parking management, and road usage pricing. The sixth factor is the technological advancement noted in recent years in various fields such as the Internet of Things (IoT), artificial intelligence (AI), computer vision (CV), and deep Learning (DL). The noticeable advancement in these areas has attracted attention. This is due to the efficiency of the algorithms, the high number of utilities, and the adaptability to different scenarios and cases. The ANPR systems represent, among others, one aspect of the global transition into smart cities’ features and applications.

However, the design and implementation of ANPR systems is a challenging problem. Although the technical advancements increase its feasibility and efficiency, many issues still need to be carefully addressed. The first problem is that, in actual cases, the images used to identify the license plate number are captured from unconstrained environments. The license recognition algorithm should efficiently recognize the plate characters regardless of the quality of the image, its resolution, the lighting conditions, and the position of the camera from the plane of the plate (distance from the camera and the translation and rotation parameters between the camera focal plane and the plane of the license plate). If needed, the ANPR system may set some constraints on the position of the license plate and the lighting conditions. However, increasing these constraints reduces the system’s portability and the ability to treat variant scenarios and cases. The second challenge met in the ANPR system is the variability in the number plate designs. The designs differ from one country to another. In the same country, one may have different designs following the plate’s creation date, the corresponding vehicle type, the driver allowed to drive it, and the location from where the plate is issued inside the country. It is recommended that the ANPR system focuses on the designs used in a specific country and solves all the related technical issues. The third challenge is the orchestration between the different sensors, the processing units, the application interface, and the cloud infrastructure to manage the application’s workflow. The license plate should be efficiently recognized in real time, and the data should be directly synchronized with the different parts of the cloud application. This is a significant concern for analyzing and managing the tracked vehicles in the system. These are the three main challenges to be solved by every ANPR system to be used in real scenarios.

In this paper, these three challenges are profoundly and consistently targeted, and a complete ANPR system for Saudi Arabian license plates is designed and assessed. Our work is the fruit of a commercial-level product tested and validated in real cases inside Prince Sultan University (PSU). Multiple tests have been performed to monitor vehicles circulating inside the PSU campus and parking entrances. The system won two international awards. The first award was granted in the KAUST Challenge for Hajj & Umrah 2020, in the Mobility track [3]. The second award was the AI Leadership Award for the best AI Product granted at the Saudi International Artificial Intelligence & Cloud Expo 2022 [4].

In this paper, we present a novel automatic number plate recognition (ANPR) solution that focuses on four key contributions made over previous works on the same problem. Our approach significantly differs from previous works as we tackle the challenge of license plate detection and recognition in real-world deployment scenarios using low-resolution video frames, whereas most studies have focused on static images. The problem we address in this paper is more challenging as it addresses real-world working scenarios These contributions are summarized as follows:

- This study proposes a novel multi-stage system for real-time vehicle identification based on deep learning models. Two different techniques (single-character and double-character detection) are presented and assessed. To the best of our knowledge, the matching post-processing procedure for the single character detection and the double character detection approach are both introduced for the first time in the literature.
- New dedicated datasets of vehicles, license plates, and license characters are carefully collected and manually labeled.
- A set of carefully designed algorithms efficiently integrate two object detectors, an image classifier, and a customized version of the DeepSort tracker, so that the system processes video streams in real-time and sends unique information about cars and license plates to a cloud server while maximizing the accuracy by taking advantage of redundancies.
- Extensive experiments are carried out on images and videos recorded under realistic conditions to evaluate the performance of the proposed system. The results show a promising performance both in terms of accuracy and inference speed, after optimizing the detection and classification models using TensorRT to run them on edge devices.

The paper is organized as follows: Section 2 will examine the research works that targeted the same problem in the literature. Then, Section 3 is dedicated to presenting the theoretical methodology of the different algorithms designed in the system. After that, in Section 4, the ANPR system is tested, and the experimental details are generated and discussed. Finally, in Section 5, the paper is concluded alongside the main limitations to solve and the possible extensions to address in future works.

## 2. Related Works

In recent years, vehicle identification and license plate recognition have gained significant interest as they represent the core technologies of intelligent transportation systems. Several works in the literature have used deep-learning-based techniques to achieve this task. Wang et al. [5] presented a comprehensive review of different types of DL-based techniques for vehicle re-identification. The authors organized these techniques into five categories: local features, representation learning, metric learning, unsupervised learning, and attention mechanism. Wang et al. compared these techniques and explored the challenges and potential research prospects for vehicle re-identification. In [6], Boukerche and Ma conducted a review of DL-based models for vision-based automated vehicle recognition (VAVR). The authors presented different vehicle recognition datasets used in VAVR, discussed the significant challenges and research trends, and summarized the characteristics of VAVR methods. Llorca et al. [7] proposed a VAVR approach based on the modeling of the geometry and appearance of car emblems from rear view images. They used HOG features and a linear SVM classifier. They reached an accuracy of 93.75% on a small dataset of 1.342 images containing 52 different car models and generations. Lee et al. [8] used a variant of the SqueezeNet architecture with bypass connections to recognize car makes and models from frontal views. They reached an accuracy of 96.3% with an inference time of 108.8 ms. For the same aim, Manzoor et al. [9] used classical random forest and SVM classifiers to obtain a maximum accuracy of 97.89% and a maximum inference speed of 35.7 images per second. The performance achieved by these models is remarkable, but none of them took advantage of the temporal redundancies in video streaming as we propose to do in this work.

On the other hand, Shashirangana et al. [10] explored the methods used in automated license plate recognition (ALPR). The authors presented recent techniques and identified the open challenges faced by the research and development community. Shashirangana et al. categorized the DL-based methods used in ALPR into two families, single-stage DL-based methods and multi-stage object detection DL-based methods. Several recent works were conducted in license plate recognition and vehicle identification. Liu et al. [11] proposed a DL-based approach to progressive vehicle re-identification called “PROVID”. The authors adopted two progressive search processes. A coarse-to-fine search is used to extract the appearance attributes using a CNN model and a near-to-distant search is used to perform a Siamese neural network-based license plate verification. Liu et al. built a dataset named VeRi-776 from urban surveillance videos. In [12], Selmi et al. developed a system for LP detection and recognition based on the DL approach, including three steps: detection, segmentation, and character recognition. Several morphological operations, such as adaptive thresholding, fine contours, and geometric filtering, are applied before applying the three previous steps. The LP detection is based on the CNN model, and the detected objects are classified into plate/no plate classes. Then, characters in upper case format (A–Z) and digits (0–9) are recognized using another CNN model with 37 classes. Kessentini et al. [13] focused on the problem of multilingual LP detection and recognition. The proposed system is based on two DL stages: (1) LP detection on raw images using YOLOv2 [14] and (2) recognition of LP on cropped images. The authors compared two recognition engines: a convolutional recurrent neural network for the whole LP recognition without prior segmentation and a joint recognition approach at the plate component level. The application of the Kessentini et al. approach was in used the Tunisian LP context. Hendry et al. [15] addressed the problem of car LP detection using YOLO [16]. The authors tweaked the original YOLO to develop a single-class detector with 36 models. The detection process is based on a sliding window for all classes in each image to avoid problems with small object detection. Their approach was applied in Taiwan’s car LP context. The proposed system was tested under different conditions, such as rain, darkness, and various visual colors and saturation.

The first work that targeted the case of the automatic recognition of Saudi License Plates (SLP) was introduced in 2003 by Sarfraz et al. [17,18]. They constructed a dataset of 610 vehicles under different illumination conditions. First, the SLP is detected using vertical edge detection, filtering, and matching. Then, the characters are extracted by counting the number of black pixels in every pixel column to extract each character apart. Finally, every character is normalized before being recognized using template matching. The association of the character to its class is based on the Hamming distance. The method is now significantly outdated now. Moreover, the license plate format used is not the standard version to consider in the current style of SLP.

In 2008, Zidouri et al. [19] proposed another pipeline. The SLP is detected using vertical edge detection by the intermediate of the Sobel filter. The detection is enhanced later by adding a thickening mask and a list of predefined conditions related to the SLP position in the image. The character segmentation is based on pixel counting for every column. Finally, neural networks (multilayer perceptrons) are used to recognize the segmented characters. They tested their method on 61 plates and successfully recognized 97% of them. More recently, some new research works focused on SLP using DL-based techniques. Khan et al. [20] presented a system for automatic LP recognition based on DL in an unconstrained environment. The authors captured real traffic videos using a mobile phone to generate their dataset of static images. YOLOv5 is used to detect the LP and a CNN model to recognize the detected alphanumerics. In order to be able to apply their method to other datasets, Khan et al. did not consider Arabic characters in the LP and only focused on English text. Consequently, they did not exploit all the information available in SLPs. Driss et al. [21] proposed a DL-based approach for Saudi Arabian LP detection and recognition. The authors developed a faster region-based convolutional neural networks (Faster-RCNN) model for detecting LP and a CNN for LP recognition. Experiments were conducted using 1150 Saudi Arabian car images that were collected under various lighting and weather conditions. The proposed system achieved a precision of 92% for LP detection and 98% for LP recognition. Table 1 compares the most recent works related to the present paper. Nevertheless, none of them directly worked on video streaming or used an object tracker in their solution.

According to most related research, working under unconstrained conditions is a major challenge for LP detection and recognition. Performances will dramatically decrease when changing the view angle, the distance between the camera and the license plate, and the light and weather conditions. The present study proposes a complete system for Saudi LP detection and recognition. Two different techniques are proposed (single character and double characters) to benefit from the redundant information in letters and digits. In addition, two YOLOv4 object detectors for cars and LP and for license characters are used. The proposed system can classify car models (196 classes) using a customized Xception. Finally, a customized version of the DeepSort multi-object tracker is developed, allowing one to send unique images of detected cars and LP to a cloud server and maximize the accuracy of the object detector and image classifier using weighted voting.

## 3. Materials and Methods

This section explains how each stage of the vehicle identification system was designed and separately assessed on validation and testing datasets in a constrained environment, and how the different models were integrated together. Subsequently, Section 4 will present the results when testing our complete system in real cases within an unconstrained environment.

### 3.1. Car and License Plate Detection

The first stage consists of detecting cars and license plates in each frame. For this aim, a dataset of 203 images of cars and license plates in a Saudi Arabian context was built, by collecting images from the Internet and photos captured by mobile phones, then manually labeling them. The images contain 819 cars and 246 license plates. In fact, license plates are often hidden by other cars, obstacles, or the car is captured from a side view. This dataset is subdivided into 90% for training and 10% for validation. Table 2 shows the number of images and instances in each set.

We trained a YOLOv4 [30] model with an input size 416 × 416 on this dataset. The choice of YOLOv4 is justified by its good trade-off between precision and inference speed [31,32], compared to other object detectors such as YOLOv3 [33], Faster R-CNN [34], or EfficientDet [35]. We used transfer learning from the COCO dataset [36] (pre-trained for 500,000 steps), a batch size of 64 images, and a learning rate of 1 × 10${}^{-3}$. After 18,900 additional steps, the model reached an average precision (AP) of 67.6% and 80.7% for the car and license plate classes, respectively, and a mean average precision (mAP) of 74.1%. All AP and mAP values presented in this paper are calculated for an Intersection over Union (IoU) threshold of 0.5 between the ground truth and the predicted bounding boxes.

**Table 2:** Number of images and instances in the training and validation datasets for car and license plate detection.

	Training Set	Validation Set	Total
Number of images	183	20	203
Number of car instances	740	79	819
Number of LP instances	227	19	246

### 3.2. Car Model Recognition

To classify car models, a dataset of 41,521 images was built by collecting images from the Internet and photos captured by mobile phones, then manually labeling them following the pattern: make–model–generation. This dataset was split into training, validation, and testing sets. For this car model classification problem, as well as for the other object detection and classification problems described below, we opted for one random train–validation–test split, since we use relatively large datasets. Whereas k-fold cross-validation approaches are preferable when dealing with small datasets [37]. Moreover, we are using the validation and testing image datasets only for an indicative evaluation of the models’ accuracy, while the final evaluation will be conducted on videos, as will be described in Section 4, since the paper’s main objective is to deal with the problem of the video streaming processing.

**Figure 1:**
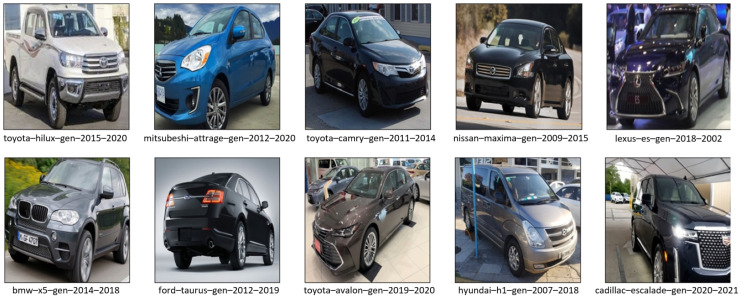
Sample images of the dataset used for car model classification (input size of 224 × 224). The labels below each image show the car’s make, model, and generation.

Table 3 shows the number and percentage of images in each set. Furthermore, Figure 1 depicts a few sample images of the dataset. It contains 24 different makes (e.g., Audi, Toyota, Hyundai, …), 90 different models (e.g., Audi Q7, Toyota Camry, Hyundai Accent, …), and 196 different generations (e.g., Audi Q7 generation 2009–2015, Toyota Camry generation 2012–2017, Hyundai Accent generation 2006–2011, …) which are considered as the final classes. We tried to keep the dataset as balanced as possible, but the way the dataset was collected caused some discrepancies, with an average of 189 images per class in the training dataset, a minimum of 73, a maximum of 384, and a standard deviation of 48.

**Figure 2:**
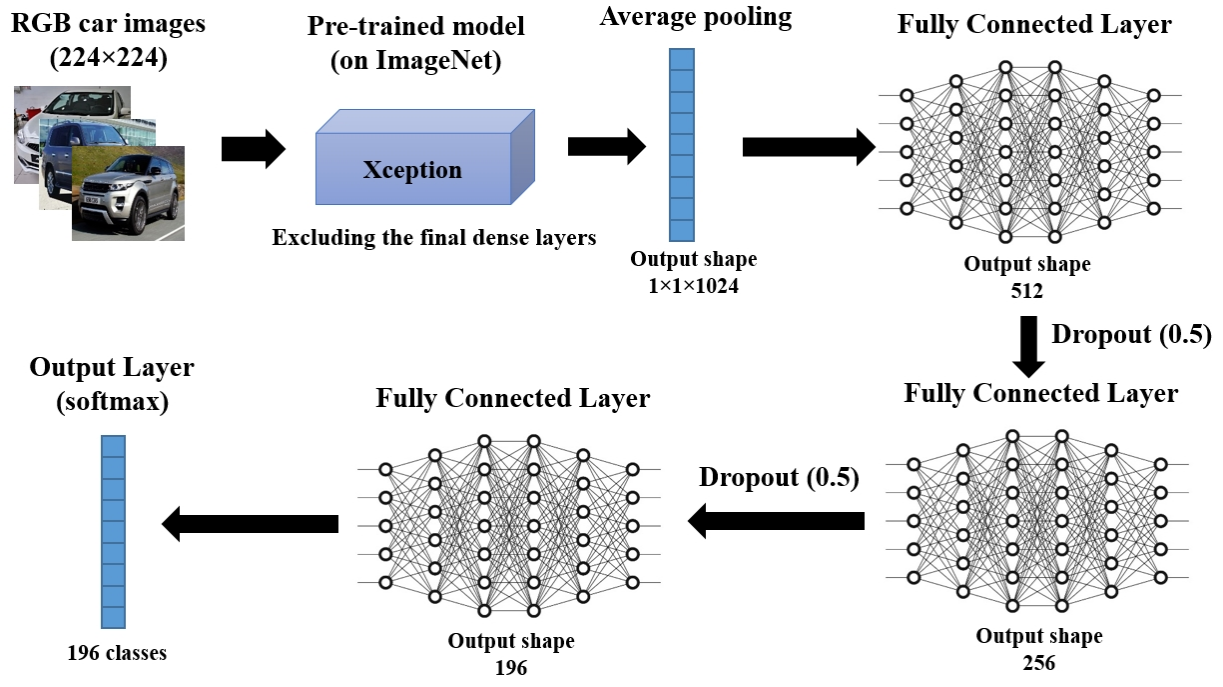
Architecture of the custom network used for car model classification.

A custom network with the Xception [38] model (pre-trained on ImageNet [39]) was used as a feature extractor. Inspired by previous tests on similar datasets, we replaced the last fully connected layer in the Xception network with an average pooling (4 × 4) and then a series of three fully connected layers followed by dropouts, and finally a softmax layer containing 196 output neurons corresponding to the 196 car model and generation classes. Figure 2 illustrates the architecture of the obtained network. The hyperparameters used for training the model are shown in Table 4.

Table 5 shows the results obtained on the testing dataset. After training for 800 epochs, the model reached a precision of 97.5% and a recall, F1-score, and accuracy of 97.3%. As an ablation test, we retrained the model after removing two fully connected layers and their subsequent dropout (at least one fully connected layer is needed to link the average pooling layer to the output layer). The accuracy decreased to 91.1%, which empirically justifies our model architecture choice.

The training took approximately 17 h on a server equipped with 8 RTX8000 GPUs. Nevertheless, the training is was only performed once, and the authors will show in Section 4 that this model along with the other detection and classification models described below runs in real-time in the inference phase.

**Table 3:** Number and percentage of images in the training, validation, and testing sets of our car model classification dataset.

	Training Set	Validation Set	Testing Set	Total
Number of images	36,953	2284	2284	41,521
Percentage	89%	5.5%	5.5%	100%

**Table 4:** Hyperparameters used for the training of the car model classifier.

Training Hyperparameter	Value
Image input size	224 × 224
Batch size	1024
Learning rate	$1\times 10^{-4}$
Optimizer	Adam
Loss function	Categorical cross entropy
Nb epochs	800

**Table 5:** Evaluation results of the car model classification model on the testing set.

	Precision	Recall	F1-Score
Macro average	97.5%	97.3%	97.3%
Weighted average	97.5%	97.3%	97.3%
Accuracy			97.3%

### 3.3. License Character Recognition

After detecting the license plate, the aim was to recognize all the characters it contains in the correct order. For this purpose, two different approaches were tested that will be described in this section after a brief introduction to the characteristics of Saudi license plates.

The two approaches that will be presented here are both based on deep-learning object detectors in their initial step. In opposition to several previous works in the literature, we did not use classical the optical character recognition (OCR) techniques for recognizing license plate characters. In fact, the preliminary tests of existing OCR tools revealed that they were highly inaccurate for Arabic characters in license plates, especially when operating in unconstrained environments. This was also confirmed in the work of Khan et al. [20] who state that “OCR could not read the number plates in any given frame”. Additionally, there is no simple way to take advantage of the redundant information in the LP if one opts for classical OCR techniques.

#### 3.3.1. Characteristics of Saudi License Plates

**Figure 3:**
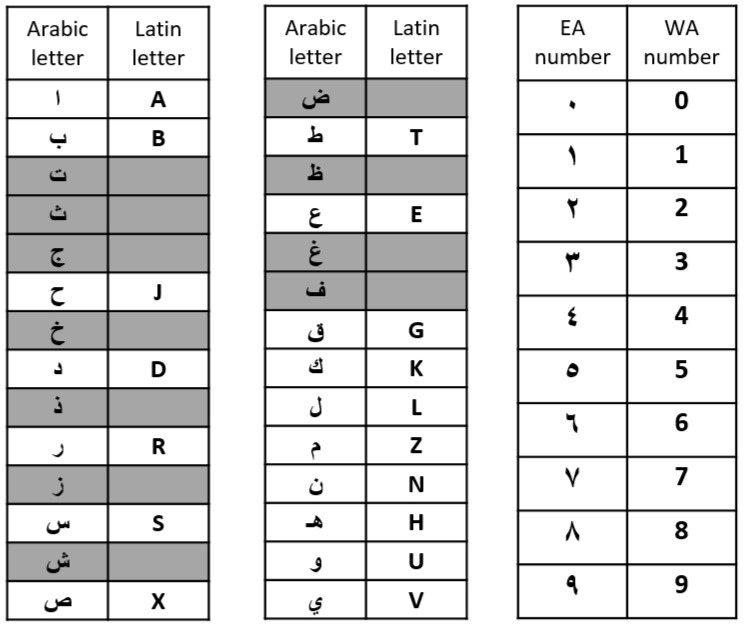
Correspondence between Arabic and Latin letters, and between Eastern Arabic (EA) and Western Arabic (WA) digits in Saudi license plates. Letters that are not allowed on Saudi license plates are shown on a gray background.

Saudi license plates are divided into four blocks: Eastern Arabic (EA) numbers—used in Eastern Arabic countries (Egypt, Gulf countries, Levant, Iraq) where they are simply called “Arabic numerals”—on the top left; the corresponding Western Arabic (WA) numbers—used in the Maghreb and a large part of the world, where they are also called “Arabic numerals” as opposed to “Hindi numerals”—on the bottom left; Arabic letters on the top right; and the corresponding Latin characters on the bottom right. In recognition of the significant historical controversy between Eastern and Western Arabic countries on which numerals are the original Arabic ones, the terminology was chosen here to avoid this controversy. The correspondence between EA and WA digits is known and straightforward. However, the correspondence between Arabic and Latin letters is specific to Saudi license plates and is not always based on phonetic similarity. Figure 3 presents this correspondence. While all ten digits can appear on Saudi license plates, this is not the case for letters. Only 17 out of 28 Arabic letters are allowed. The remaining letters were discarded by the Saudi traffic authorities because of their high similarity to the included letters. This makes a total of 54 possible characters. The one-to-one correspondence between characters on the first and second lines of Saudi license plates offers redundant information that will be exploited in the proposed license character recognition system. Latin letters and WA digits are always placed exactly under their corresponding Arabic letters and EA digits on SLPs, even though the Arabic language is normally written from right to left.

**Figure 4:**
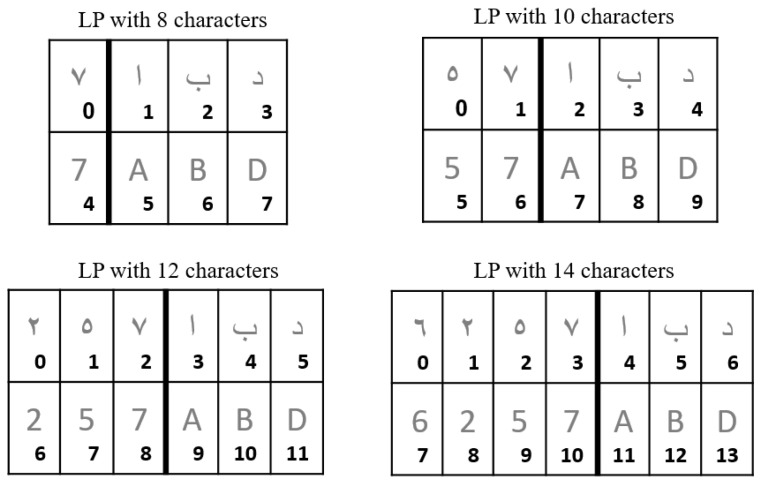
Example showing the possible number of characters on Saudi license plates. There are always three letters, but the number of digits varies from 1 to 4. Each letter is written in both Arabic (first line) and Latin (second line) characters, and each digit is written in Eastern Arabic (first line) and Western Arabic (second line) numerals. For later use, each character is marked with an index (CharID) representing its order from left to right and top to bottom.

**Figure 5:**
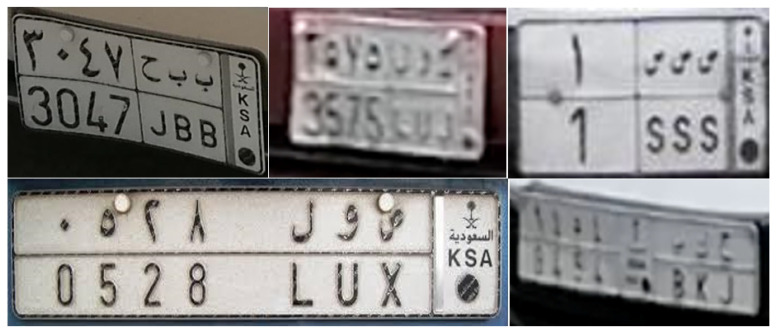
Sample images from the dataset used for single-character detection showing images of different quality and angle views and two different aspect ratios of Saudi license plates using different fonts. We intentionally kept low resolution images, considering that this is the type of resolution expected in real deployment.

On the other hand, there are complicating factors for the automatic recognition of Saudi license plates. First, the number of characters is variable. The plate always contains three letters, but the number of digits (for EA and WA numerals each) ranges from 1 to 4 (Figure 4), which means that the total number of characters on an SLP can be 8, 10, 12, or 14. Second, there are two different shapes of Saudi license plates still in use today, each using a different font and different space size between characters (Figure 5). One shape has a horizontal and a vertical line delimiting the four blocks of characters, while the other has only a horizontal line. Therefore, these edges cannot be reliably used to segment SLPs.

#### 3.3.2. First Approach: Single-Character Detection

**Figure 6:**
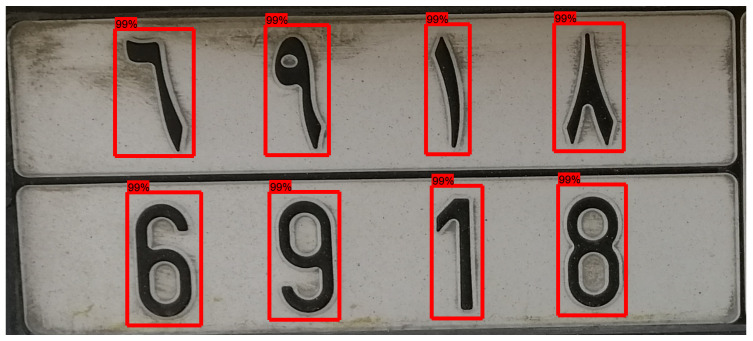
Example of single-character detection on a license plate.

The first approach that is implemented here consists of applying an object detector to extract each character separately (character segmentation, Figure 6), then applying an image classifier to recognize the character. Finally, a matching procedure is used to enforce that the two lines of characters are consistent and to leverage the redundant information they contain.

**Character detection:** For single-character detection, we built a dataset of 373 cropped images of Saudi license plates with variable image quality, resolution, and inclinations. A small sample of these images is presented in Figure 5. These images were manually labeled by defining an accurate bounding box delimiting every single character in the license plates. The dataset is subdivided into training (90%) and validation (10%) sets. Table 6 shows the number of images and character instances in these two sets. The number of characters per license plate varies from 8 to 14, as explained in Section 3.3.1, but license plates with 14 characters are largely the most common. This explains why the average number of characters per license plate in our dataset is 13.9.

We trained a YOLOv3 model (pre-trained on the COCO dataset) on our dataset, with an input size of 320 × 320, a batch size of 64, and a learning rate of $1\times 10^{-3}$. The choice of YOLOv3 (instead of YOLOv4) with a reduced input size is justified by the necessity for a lightweight and faster object detector since it will have to detect up to 14 characters on each license plate. After 39,000 steps of training, the model reached an mAP of 99.8% on the validation set. This level of accuracy shows that it is needless to use a more complex object detector for this task, at least on the current dataset.

**Table 6:** Number of images and instances in the single-license-character detection dataset.

	Training Set	Validation Set	Total
Number of images	336	37	373
Number of character instances	4678	515	5193

**Character classification:** After detecting and extracting every single character in the license plate, they are fed to an image classifier to recognize them. Since up to 14 characters need to be recognized for each license plate, we opted for MobileNetV2 [40], which is a lightweight CNN with 3.5 M parameters, as a feature extractor that is pre-trained on ImageNet. We adopted an architecture similar to the one we used for car model classification (Figure 2) by replacing the last fully connected layer in the MobileNetV2 network by an average pooling (4 × 4) followed by a series of 3 fully connected layers followed by dropouts, and finally, a softmax layer containing 54 output neurons corresponding to the 54 character classes.

We collected a dataset of license plate characters by extracting cropped character images from the above-described license plate dataset (Table 6), to which we added thousands of other cropped characters obtained by running the character detector on images and the videos of cars. Then, we manually labeled all the extracted characters. The total number of characters thus obtained is 18,422, which was subdivided into a training set (95%) and a validation set (5%). All character images were resized to 128 × 128, to be processed by the custom MobileNetV2-based CNN network described above. After training for 400 epochs, the model reached a validation accuracy of 99.4%.

**Figure 7:**
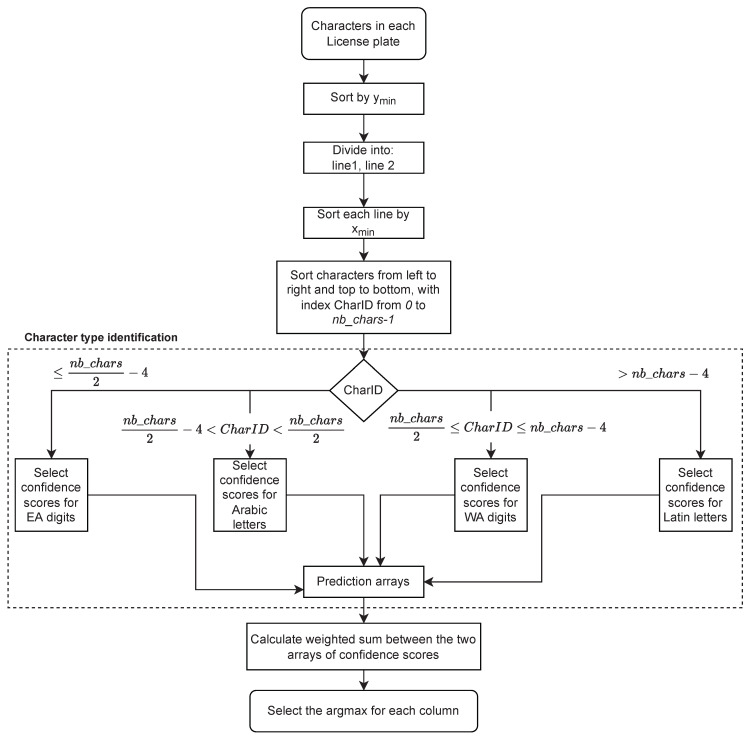
License plate character identification for each frame, in the case of single-character detection. EA stands for Eastern Arabic, while WA stands for Western Arabic.

**Matching procedure:** After detecting each license character separately, a matching procedure is applied to enforce that the two lines of characters are consistent with each other, and to take advantage of the redundant information that they contain. Figure 7 illustrates the post-processing procedure for ordering license plate characters and selecting them based on the maximum confidence yielded by the character classifier, subject to the constraints of the position of letters and digits on Saudi license plates (as can be seen in Section 3.3.1). The character bounding boxes are first sorted with respect to $y_{min}$, which corresponds to the vertical position of the top of the bounding box in the frame. Based on this order, the set of characters is divided into two lines. Then, the characters of each line are sorted with respect to $x_{min}$, which corresponds to the horizontal position of the bounding box’s right side. The characters thus ordered from top to bottom and from left to right are assigned an index called charID ranging from 0 to nb_chars−1, where nb_chars is the number of detected characters (as can be seen in Figure 4).

**Figure 8:**
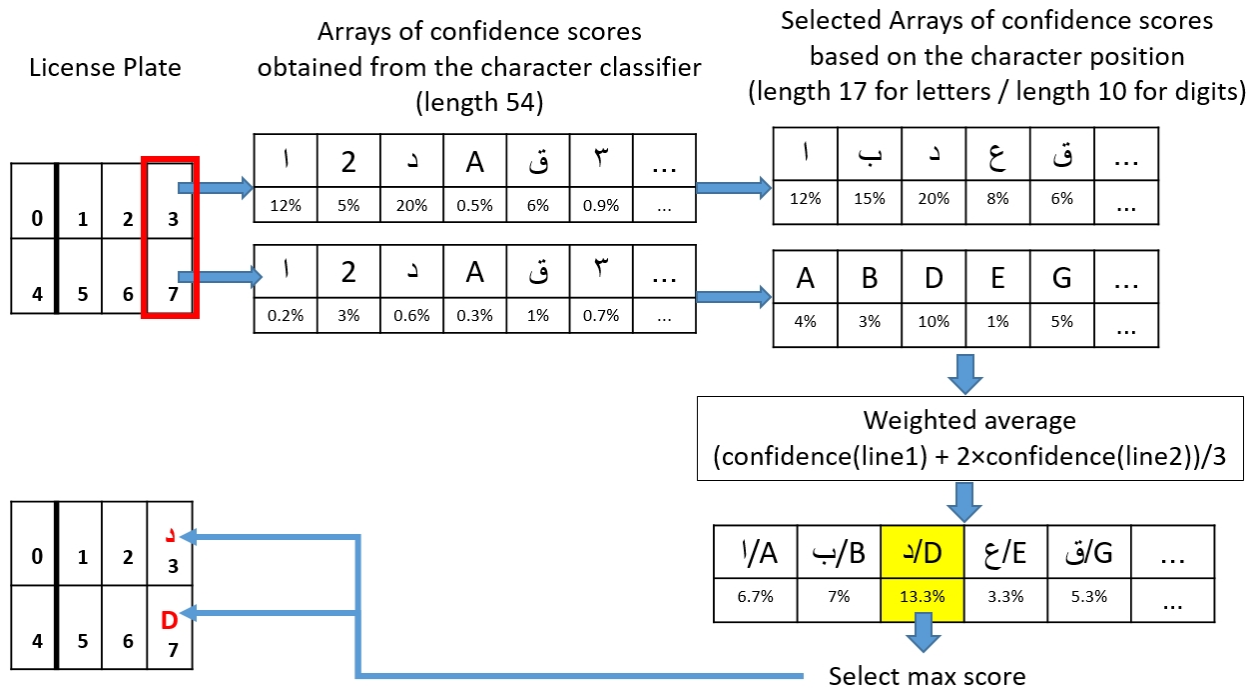
Post-processing of the character classifier output in the single-character detection approach.

The output of the character classifier is a vector of length 54 (number of character classes) containing the confidence score of each class. From the value of charID, the system infers the type of character (Western Arabic digit, Eastern Arabic digit, Latin letter, Arabic letter), and only keeps the classes corresponding to the inferred type, with their associated confidence scores (Figure 8). Then, for each character position, we apply a weighted average between the array of confidence scores in the first line and the array of confidence scores in the second line.

We noticed a higher rate of confusion between the characters of the first line (Arabic letters and Easter Arabic digits) than between the second line of characters (Latin letters and Western Arabic digits). This is due to greater shape similarities between the characters of the first type. Based on preliminary results, we empirically fixed a weight of $\frac{1}{3}$ for the first line’s confidence scores and $\frac{2}{3}$ for the second line’s confidence scores. Then, for each two-character column in the license plate, the pair of characters that corresponds to the maximum weighted average of confidence scores was selected. This will be the selected pair of characters for the given frame. Ultimately, voting will be applied on a series of frames to select the final characters for the given license plate, as described in Section 3.6.

#### 3.3.3. Second Approach: Double-Character Detection

**Figure 9:**
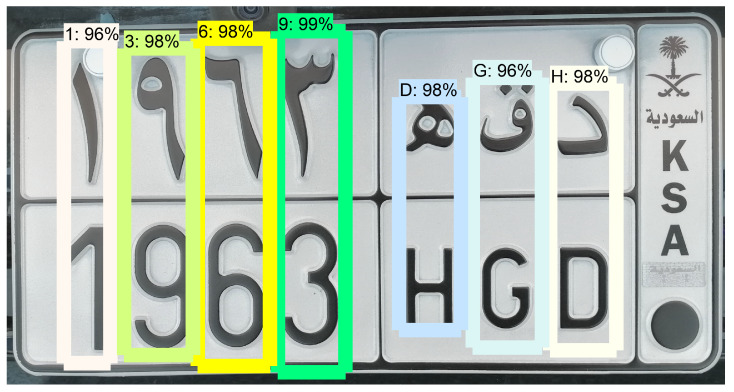
Example of double-character detection on a test image.

The second approach that we tested for character recognition consists of training an object detector to recognize the double characters (one character on the first line and the corresponding character on the second line), as depicted in Figure 9. The advantage of using this approach is to increase the distance between class features, making them more discernible and eliminating the need for complex post-processing matching. Additionally, instead of using a one-class character detection followed by image classification as in the first approach, this second approach opts for a one-stage 27-class character detection. For this aim, we collected a dataset of 593 license plate images that we divided into training (95%) and validation (5%) sets. Table 7 shows the number of images and instances in each set. The dataset was not exactly balanced, since some characters appear more frequently on Saudi LPs. The average number of images per class is 142, the standard deviation is 84, the minimum is 37 (for character ‘U’), and the maximum is 276 (for number ‘7’). The number of double characters per license plate ranges from 4 to 7 (see Section 3.3.1). However, since Saudi license plates with seven double characters are the most common, the average number of double characters per license plate in our dataset is 6.5.

A Yolov4 model was trained on this dataset, with an input size 416 × 416, pre-trained on the COCO dataset. We opted for YOLOv4, compared to YOLOv3 that we used for the first approach, and a larger input size because the task of distinguishing between 27 classes is more complex than the mere detection of a character. Additionally, the number of characters to be detected per license plate is 7 (in most cases) compared to 14 for the first approach. Moreover, this second approach eliminates the need for an image classifier since character detection and classification are now merged into one model. For all these reasons, there is more room for using a more sophisticated model and a larger number of network parameters.

After only 5000 training steps, the model reached an mAP of 99.0% on the validation dataset, with an AP per class ranging from 91.7% (for the letter H) to 100% (for 21 out of 27 classes). Once double characters are separately detected and recognized in a given license plate, in order to obtain the correct order of characters, the system only needs to sort their bounding boxes according to their position on the x axis, instead of the complex matching procedure that was used in the first approach.

**Table 7:** Number of images and instances in the double-character detection dataset.

	Training Set	Validation Set	Total
Number of images	561	32	593
Number of character instances	3628	199	3827

### 3.4. Tracking

Since our objective is to recognize car models and license plates from a video stream, not from a single image, the system needs to know whether the object detected in a given frame is the same as the object detected in previous or subsequent frames so that the recognized car model and plate are sent to the server only once. To this aim, we integrated a tracker with the system. We opted for DeepSORT [41] which is a real-time multi-object tracker that extends simple online and real-time tracking (SORT) [42] by adding a pre-trained deep association metric that incorporates object appearance information.

**Figure 10:**
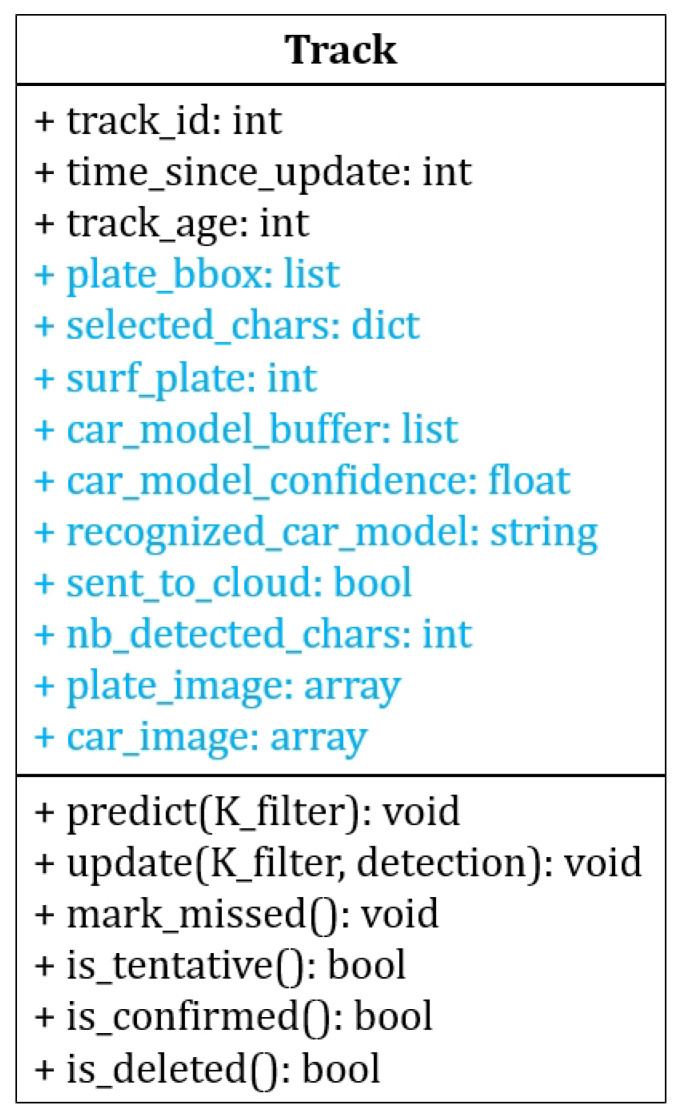
UML diagram of the Track class. The original DeepSort attributes and methods are shown in black, while the attributes that we added to integrate the tracker with our system are shown in blue.

**Figure 11:**
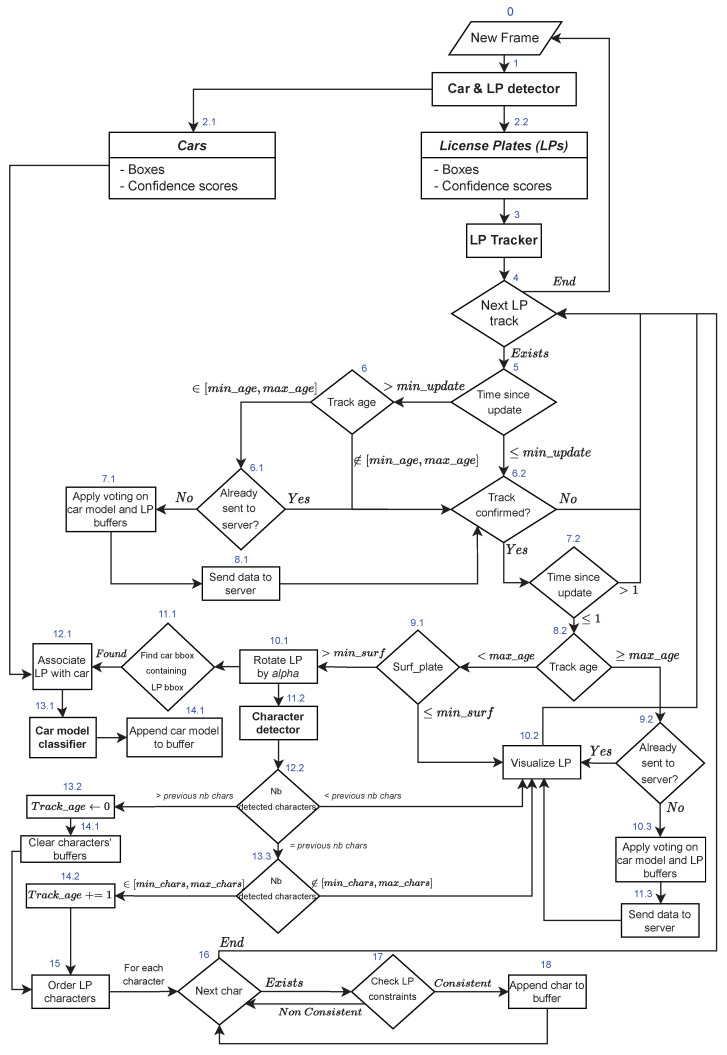
Complete process of the car model classification and license plate recognition. Step numbers are shown in blue.

To minimize computations, the DeepSORT tracker is only applied to license plates, not to cars. The corresponding car is inferred from the relative position of the car and license plate bounding boxes. To be able to integrate the DeepSort tracker with our system, we customized it by adding several attributes of the detected car and its license plate to DeepSORT’s track class, as shown in the UML diagram in Figure 10:

- plate_bbox: a list containing the detected LP bounding box coordinates on the image.
- selected_chars: a dictionary containing a list of selected characters for each license plate position. The length of the list corresponds to the number of frames in which the character was detected.
- surf_plate: surface of the detected license plate, in pixels (width×height).
- car_model_buffer: a list containing the car model yielded by the classifier for each frame in which the car was detected.
- car_model_confidence: a list containing the car model’s confidence score for each frame in which the car was detected.
- recognized_car_model: name of the currently selected car model, after voting on the series of frames in which the car was detected. This name is visualized in real-time, but the definitive name will only be sent to the server with the conditions defined in Figure 11.
- sent_to_cloud: Boolean variable indicating whether the information of the track was already sent to the server or not.
- nb_detected_chars: number of detected characters in the license plate for the current frame.
- plate_image: license plate image saved as a Numpy array. When the same license plate (same track_id) is detected in a series of frames, only the image having the maximum surface is saved.
- car_image: car image saved as a Numpy array. When the same car is detected in a series of frames, only the image having the maximum surface is saved.

The way these attributes are used will be explained in more detail in Section 3.7.

### 3.5. Model Optimization

The original object detection and classification models that we designed in our system cannot be run together on edge devices with limited memory and computing capacities, such as Jetson boards. Therefore, to be able to run the system on a Jeston Xavier AGX edge device, all these models have to be optimized using NVIDIA TensorRT framework. TensorRT applies various optimization operations specifically dedicated to the target device. This includes quantization, which consists of reducing the precision of weight values and activation functions with minimum accuracy loss. It also performs layer and tensor fusion by fusing different nodes to optimize memory and bandwidth GPU usages. Previous works [43] have shown that TensorRT-optimized deep learning models provide the fastest execution on a wide range of cloud and edge devices compared to other types of optimization such as TFLite, with a difference in inference speed that reaches 62%.

### 3.6. Edge-Cloud Communication

Our system is coupled with an end-user full-stack web interface installed on a cloud server that enables the manipulation of the collected data and search of vehicles based on their features and license plates. Basically, for each detected and tracked car, a message is sent from the edge to the cloud server only once, when one of the two following conditions are fulfilled:

- The car quits the camera field of view for a predefined number of successive frames (min_update);
- The car has been tracked for a predefined number of successive frames (max_age).

After the data are sent, the car model recognizer and the license character detector are no longer applied to the identified object (which is still in the camera field of view or quit and then reappeared with the same tracking ID). However, the tracker is still applied since the system needs to know whether the detected object is a car whose information has already been sent to the server or a car that is detected for the first time. The detailed conditions for sending the car information to the server will be detailed in Section 3.7 and Figure 11.

For each tracked car, the recognition results of car models and series of license characters in each frame are kept in a list. When the information is ready to be sent to the server, a weighted vote is applied on each list to select the best guess. Based on preliminary tests, we set the weights as the confidence scores for car models, while for license plates, we set them as the license plate surface in each frame. This is because, in most cases, the larger the license plate (i.e., the closer to the camera), the better the detection and recognition of license characters. Whereas for car models, the authors noticed that this is not the case. This is likely due to the aspect of the cars in the training dataset.

Each message sent to the server contains the following information:

- The car cropped image (in base 64).
- The license plate cropped image (in base 64).
- The predicted car model.
- The car model confidence (averaged on a series of frames).
- The predicted license plate.
- The license plate confidence (averaged on a series of frames).
- The timestamp.
- The camera ID and location.

**Figure 12:**
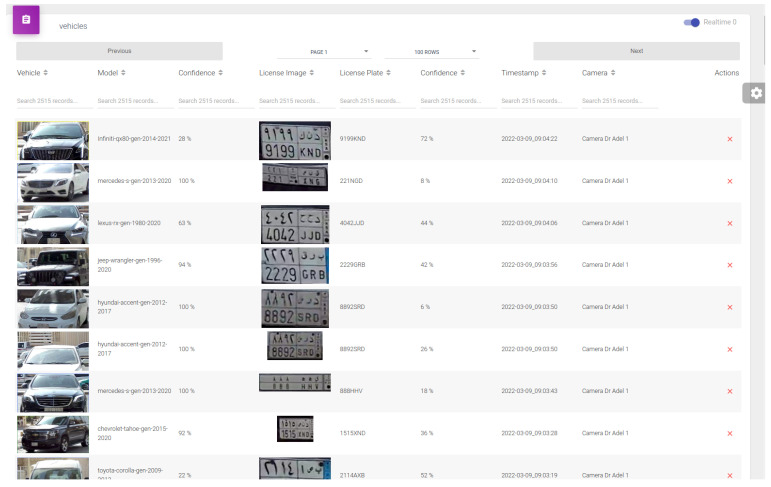
Screenshot of the vehicle web interface that displays car information received from the edge device.

When the server receives the message, it is stored in the dataset and displayed in real-time on the web interface. Figure 12 shows a sample screenshot of the web interface installed on the server.

### 3.7. Description of the Complete Multi-Stage Process

Figure 11 shows the flowchart of the complete process of the proposed multi-stage car and license plate recognition system. For each new frame, the car and license plate YOLOv4 detector is first applied (step 1 in Figure 11). It yields a list of bounding boxes and confidence scores for both cars and license plates (steps 2.1 and 2.2). A score threshold is applied (0.2 as the default value) before the DeepSort tracker is fed with detected license plate bounding boxes (step 3) to be matched with the existing tracks. The tracker returns pairs of matched tracks and detections, a list of unmatched tracks, and a list of unmatched detections.

Subsequently, each track is examined to determine whether to send its data to the server, process them further, or discard them. If the track has not been updated with new detected bounding boxes for a number of frames higher than a fixed threshold (default value: 10), this indicates that the object has presumably left the field of view of the camera. The system checks (6) whether the tracking age (number of frames in which the same object has been observed) is in the desired interval [min_age,max_age] (fixed by default to (2,50)). A low value (<min_age) means that the results of the detection and recognition of the object cannot be sufficiently trusted enough, while a large value (>max_age) means that the object properties have already been sent to the server. Even when the tracking age is in the desired interval, the object could have been sent to the server in case it disappeared from the field of view and then returned shortly. This is why this condition is checked in step 6.1. If it has never been sent, the voting procedure for car models and license characters is applied, and then the results are sent to the server, as explained in Section 3.6.

In all cases, the system checks whether the track is confirmed (step 6.2). A track is considered confirmed when enough evidence has been collected that it is an active track observed in a minimum number of consecutive frames (3 by default) and that it has not been deleted due to repeated misses. If the track is not confirmed or has not been updated (i.e., matched with detected bounding boxes) for at least two frames, no further track processing is needed, so the system moves on to the processing of the next track. On the other hand, if the track is confirmed and has been updated in the current or previous frames, the system checks its tracking age (step 8.2). If it is larger than or equal to max_age, its attributes are sent to the server (after applying voting) if it has not been sent yet. Then, the license plate is visualized (10.2) using the current attributes of the track instance (see Figure 10). This step is optional and it is the last step in the processing of any confirmed and updated track. If the tracking age is still less than max_age, the system checks the license plate surface in pixels (9.1). If it is too small, it is just displayed without further processing since the character recognition is expected to perform poorly in such a case. The surface threshold is a parameter to be fixed depending on the camera position and the image resolution. If the license plate surface is larger than the threshold, the more elaborate processing of the track begins.

First, a rotation is applied to the image of the detected license plate (10.1). This may be needed when the camera view angle is tilted. Otherwise, the rotation angle α is fixed to 0. Then, the system searches for the car corresponding to the detected license plate (11.1). If there are no false-positive detections, and the camera is placed so as to capture only front or rear car views, there should be only one car bounding box containing the considered license plate bounding box. If it is found, it is associated with the license plate (12.1), then we apply the car model classifier (13.1) and append the result to the car model buffer (14.1) in the attributes of the license plate instance (as can be seen in Figure 10). If no corresponding car is found until the final result is about to be sent to the server, a default bounding box is cropped around the license plate to approximate the car bounding box.

In parallel, the character detection model is applied to the license plate image (11.2). Due to detection errors (false positives or—more commonly—false negatives), the number of detected characters may vary for the same license plate. Since the system only checks the relative character positions, any change in this number would conflate neighbor characters. An alternative would be to check the character positions relative to the license plate. However, this approach is more error-prone since Saudi license plates have different shapes and the space before, between, and after characters is variable (see Section 3.3.1 and Figure 5). Therefore, we decided that if the number of detected characters increases for the same license plate, relative to the previous frame, we reinitialize the buffer of characters on which voting will be applied, and then we process the characters further as will be described. If the number of detected characters decreases, it is likely due to some characters being missed in the current frame while they were detected in the previous frame. Consequently, the system dismisses the result of the character detector for the current frame, without reinitializing the buffer of characters, since the missed character(s) may be detected again in the following frames. If the number of detected characters remains the same as in the last frame and this number is in the acceptable range (between 8 and 14 if we deal with single characters, or between 4 and 7 if we deal with double characters), the system increments the tracking age of the track instance (14.2) and orders the character bounding boxes (step 15). In the case of single-character detection, this ordering follows the procedure in Figure 7. Whereas in the case of double-character detection, the system only sorts the character bounding boxes from left to right, then checks the consistency of each character (step 17) with respect to its position. The constraint is that the three rightmost characters must be letters (as can be seen in Section 3.3.1). If the constraint is respected, the character is appended to the character buffer (18), on which voting will be applied, as explained in Section 3.6.

## 4. Results and Discussion

In Section 3, the authors presented the assessment results of each component separately on the testing datasets of images that were randomly split from the same domain as the training datasets. In the present section, the results of each stage are assessed and discussed, as part of the integrated vehicle identification system, on realistic images and videos that were captured while testing the system in different locations at PSU campus.

### 4.1. Evaluation Approach

**Figure 13:**
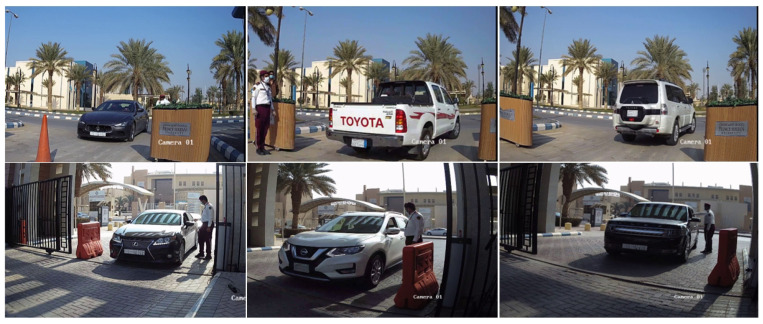
Sample images extracted from two videos for evaluating car model and license plate recognition. The images at the top are from video 1, which was recorded at the employees’ parking lot entrance, while the images at the bottom are from video 2, which was recorded at the main gate of PSU campus. As can be observed in this sample, the license plate characters are hardly readable on some frames.

We first evaluated our solution on a set of 100 images extracted from two videos recorded at different locations inside PSU campus, then on the complete videos. The authors manually labeled the videos by noting for each car the time it entered the camera field of view, the time it left it, its model and generation, and its license plate. The two videos have a total time of 32 min and 46 s, while the 100 images were extracted at fixed intervals, and show cars at variable distances from the camera and various view angles, both from the front and rear view. Figure 13 shows a sample of the images that were extracted from the two videos. The evaluation on static images mainly aims to compare the performance of the two character detection approaches presented in Section 3.3.2 and Section 3.3.3 without including the tracker which is applied in the same manner in the two approaches. The two types of evaluations will also assess the value-added of the tracker in terms of recognition accuracy for both car models and license plates. We run the system online (by connecting the edge device to the camera using RTSP stream) to ensure it runs in real-time with no delays, then offline on the two recorded videos to test the different configurations.

### 4.2. Car Model Recognition

Table 8 shows the evaluation results of the car recognition model on the set of images described above. Among the cars, 14% had models that the authors did not include in our learning dataset, so they obviously could not be correctly recognized. Among the cars, 15% were not detected by the car and license YOLOv4 detector on the considered frame. When using the whole sequence of frames in the videos, this rate drops to 1.7% (Table 9). In fact, it is sufficient to detect the car in some of the frames where it appears. This highlights the importance of using a tracker in the system. Among the cars, 5% are both non-detected and have unknown models, whilst 38% of car models (without considering generations) and 34% of the car models and generations were correctly classified. If one discards missed detections and non-included car models, these two rates rise to 50% and 45%, respectively. When compared to the 97.3% accuracy obtained on the testing dataset (which was randomly split from the same domain as the training dataset, as explained in Section 3.2), this confirms that high accuracy on a particular domain under constrained conditions does not guarantee a high accuracy on other domains with unconstrained conditions (different camera, various lighting conditions, distance to the camera, view angles, occlusions, noise due to inaccurate bounding boxes provided by the car detector, etc.). This is a well-known problem that is a corollary of the no free lunch theorem in inductive reasoning [44]. This problem can be mitigated using a much larger learning dataset, collecting a new dataset from the same domain and conditions as for actual deployment, or using domain adaptation techniques [45,46,47].

### 4.3. License Plate Recognition

Table 9 compares the two license plate recognition approaches that were described in Section 3.3.2 and Section 3.3.3, by evaluating them on the set of images described in Section 4.1. The first approach (Model 1) uses a YOLOv3 1-class single character detector (with an input size 320 × 320) followed by a custom 54-class image classification network based on MobileNetV2. Whereas the second approach (Model 2b) only uses a YOLOv4 27-class character detector (with an input size 416 × 416). To measure the influence of the input size on the accuracy and inference speed, the authors also tested the second approach with an input size of 320 × 320 (Model 2a).

The total number of characters in the testing dataset is 1382. The percentage of missed license plates is either due to a non-detection of the license plate itself or to a number of detected characters in the license plate outside the fixed range of 8–14 characters (or 4–7 double characters for the second approach). The authors imposed this constraint to discard false-positive license plates and characters, and to make only consistent predictions of full LPs. The 1-char detection approach gives a slightly better percentage of missed LPs (18%). This can be explained by the fact that when the LP is excessively tilted, it becomes more challenging to delimit double characters in straight bounding boxes (and therefore harder for YOLO to detect them) than to delimit single characters.

Model 2b yields the best accuracy for both the recognition of characters and the recognition of full LPs. When we do not impose a minimum number of detected characters per license plate, the percentage of missed LPs for Model 2b drops from 21% to 14%, but the number of correctly recognized characters also decreases from 92.5% to 87.7%, and the number of correctly recognized LPs expectedly stays unchanged at 53%. The accuracy of full LP recognition is noticeably lower than the accuracy of character recognition because the correct recognition of an LP requires the correct recognition of all the characters it contains. Thus, theoretically, if the probability of correctly recognizing one double-character is 92.5%, the probability of recognizing a seven-double-character LP is ${\left(0.925\right)}^{7}=0.579$. The discrepancy between character recognition accuracy and complete LP recognition accuracy is lower for 1-char detection than for 2-char detection, which indicates that the matching procedure defined in Section 3.3.2 is efficient and is able to compensate for the lower accuracy in character recognition accuracy.

In terms of inference speed, the 2-char detection is approximately twice as fast as the 1-char detection since it consists of only one stage (no image classifier), has fewer outputs (27 instead of 54), and requires fewer post-processing computations. Increasing the image input size from 320 × 320 (Model 2a) to 416 × 416 (Model 2b) significantly improves the accuracy of character and LP recognition with only a slight disadvantage in terms of speed. Therefore, we selected Model 2b as the best solution for character recognition, and we will focus on the evaluation of this model when integrated with the other components of the system, for the remaining time of this section.

**Figure 14:**
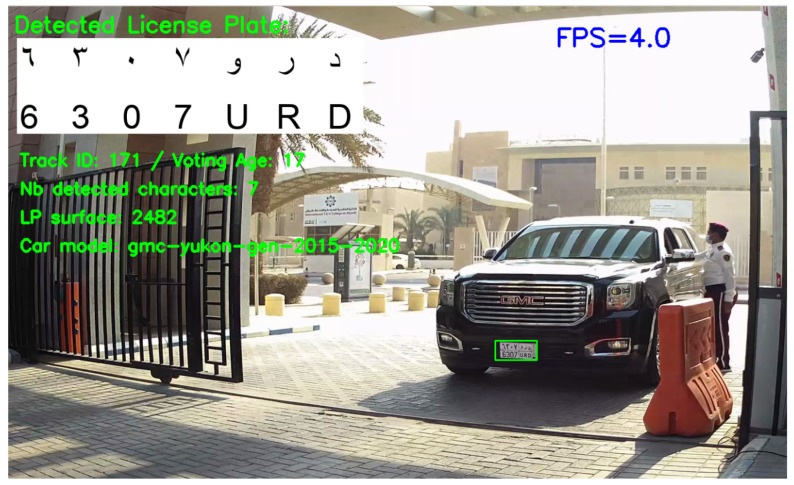
Sample frame from the processing of video 2. This online visualization is only used for checking and debugging purposes. Otherwise, it is disabled, because it significantly slows down the program, as the instantaneous FPS value on the top right reveals.

**Figure 15:**
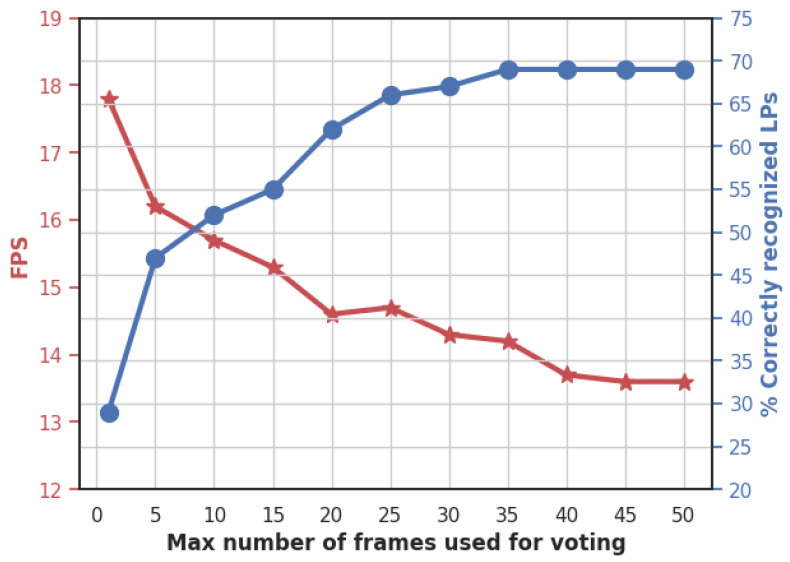
Evolution of the percentage of correctly recognized license plates and inference speed (in frames per second) in Video 1, with respect to the maximum number of frames used for voting.

After having evaluated the car model and license plate recognition on a set of static images, we evaluated the whole system (including the multi-object tracker and the communication with the cloud server) on two HD videos recorded in two different locations on the PSU campus. Figure 14 shows a sample frame from the processing of video 2, and Table 10 shows the characteristics of each video and the evaluation results. The results are better on video 2 (main entrance) because we could place the camera in a suitable position with a better view angle than in video 1. This stresses the importance of camera calibration for real deployment. Over the total of the two videos, only 1.7% of cars and 2.5% of license plates are non-detected. This is noticeably lower than the rates that were obtained on the set of still images (Table 8 and Table 9). This improvement highlights the benefit of including a tracker in the system so that even if the car or license plate is missed in some frames, they are detected in other frames. The percentage of correctly predicted characters over the two videos is 88.6%, which is lower than what was obtained on still images (Table 9, Model 2b), but the percentage of correct LPs jumps from 53% to 74% (40% relative increase) due to the use of voting on the sequence of frames where the LP appears, as explained in Section 3.6 and Section 3.7. In order to more precisely quantify the influence of voting on the recognition of LPs, we tested several values for the parameter max_age which corresponds to the maximum number of frames used for voting. Figure 15 depicts the evolution of LP recognition accuracy and the inference speed for different values of max_age when tested on Video 1. The accuracy jumps from 29% when using a single frame to 69% when using a maximum of 35 frames for each license plate. This highlights the importance of the voting process that we introduced for enhancing LP recognition by taking advantage of the temporal redundancies. The LP recognition accuracy stagnates for max_age higher than 35, which indicates that there is no need for setting high values for the max_age parameter. Additionally, the evolution of the inference speed shows that a reduced value for max_age increases the system’s average speed, since the character detector model is no longer applied when the number of frames reaches max_age (see Figure 11, step 8.2). However, the inference speed also stagnates for large values of max_age. When moving from 1 to 20 frames, the inference speed decreases by 18%, while from 20 to 50 frames, it only decreases by 7%.

On the other hand, the recognition of car models improves only slightly on the two videos compared to Table 9: from 38% to 43% (13% increase) for models, and from 34% to 37% (9% increase) for models and generations. This can be explained by the greater number of classes in the car model classifier (196 classes compared to 27 character classes), which makes the voting procedure less efficient because a given car model prediction is less likely to be repeated in different frames. Nevertheless, as already mentioned in Section 4.2, the tracker helped reduce the miss rate for car detection from 15% on static images to 1.7% on videos.

If one discards nonexistent car models, the percentage of correct classifications becomes 58% for models and 50% for models and generations. Furthermore, no false positives for cars or license plates were sent to the server for either video. Actually, the car and license plate detector produces some false positives, but cars that do not contain a license plate or plates that do not contain a minimum number of license characters inside their bounding boxes are later discarded in the processing.

In terms of speed, the average FPS on the two videos is 17.1, which is near real time. It is higher than the value shown in Table 9 because frames that do not contain cars or license plates in the videos are processed much faster. Additionally, the frames are read in a separate thread and are automatically skipped when necessary to keep up with real-time processing. Thus, no delay is accumulated, and the results are sent to the server in real time.

We also assessed the influence of video resolution by downgrading the resolution of Video 2 from 1920 × 1080 (1080p) to 1280 × 720 (720p), then to 720 × 480 (480p). Table 11 shows the results obtained for each resolution. The detection and recognition rates decrease moderately when passing to 720 p resolution, but markedly deteriorate when passing to 720 p resolution, down to 23% of correctly identified LPs. This highlights the importance of image quality when dealing with video streams. On the other hand, the inference speed only marginally increases by 6% when passing from 1080p to 720p, and by 9% when passing from 720p to 480p, which does not compensate for the loss in accuracy.

**Table 10:** Evaluation of the car and license plate recognition system (using Model 2b for character recognition) on two videos recorded under realistic conditions on the PSU campus.

	Video 1	Video 2	Total
Duration	11 mn:18 s	21 mn:28 s	32 mn:46 s
Original FPS	25	30	–
Number of unique cars	58	61	119
Number of character pairs	403	420	823
Missed LP detections	5%	0%	2.5%
Correctly predicted characters	81.9%	95%	88.6%
Correctly predicted LPs	67%	80%	74%
Missed car detections	3%	0%	1.7%
Nonexistent car models (not included in the training dataset)	24%	28%	26%
Misclassified car models	41%	23%	32%
Misclassified car models and generations	43%	32%	38%
Correctly classified car models	33%	49%	43%
Correctly classified car models and generations	31%	45%	37%
Average processing speed (in FPS)	14.4	18.4	17.1

**Table 11:** Car and license plate recognition results per different resolutions of Video 2.

Resolution	1920 × 1080	1280 × 720	720 × 480
Missed LP detections	0%	2%	20%
Correctly predicted characters	95%	91.2%	79.3%
Correctly predicted LPs	80%	72%	23%
Average processing speed (in FPS)	18.4	19.5	21.2

## 5. Conclusions

The authors designed and assessed a complete multi-stage vehicle identification and license plate recognition ecosystem in the Saudi Arabian context. It takes advantage of the recent progress achieved in deep-learning-based object detectors, classifiers, and trackers. The object detection and image classification models were trained on carefully collected and manually labeled datasets of vehicles, license plates, and license characters. The customized multi-object tracker is used to enhance the recognition accuracy and to save each car’s information only once. Even though the system involves four different deep-learning models running concurrently and sending the results to the cloud, it is able to run in real-time on edge devices with limited computing capabilities, such as Jetson boards. Under realistic testing conditions, we have proven the efficiency of our multi-stage framework.

Nevertheless, there is still a significant gap between the accuracy achieved on validation datasets in a constrained environment and the accuracy obtained in realistic unconstrained environments. This gap highlights the limitations of a large part of related works in the literature that only present results under constrained conditions. To bridge this gap, the detection, classification, and tracking models all need to be enhanced by training them on larger, more realistic datasets, along with hyperparameter optimization before the system is deployed in an industrial context. Additionally, the car model recognition results could be mapped with those of the license plate recognition to enhance them both, provided that a dataset of vehicle information is available beforehand, such as when using the system for automatic private parking entry. However, the authors did not include this assumption in the present work. Moreover, three additional challenges need to be targeted in future extensions. The first is to enhance video streaming quality using recent automatic methods (such as [48]) that address the problem of hazing due to the presence of dust, smoke, and other particles in the atmosphere that often damage the image quality. Additionally, we will assess the influence of image quality on recognition accuracy using objective video quality assessment techniques [49,50], especially those dedicated to automatic license plate recognition tasks [51,52,53,54]. This will help define the best configuration and video streaming parameters to optimize the system accuracy, reliability, and robustness. The second is securing the identity and the privacy of license plates over multiple parts of the system [55,56,57]. The third is protecting the ANPR system from forgery. In fact, fake license plates could be used for malicious purposes such as granting unauthorized access, hiding identity, or falsely accusing innocent victims. The image forgery problem in the license plate case is more complex than in other cases such as face images or fingerprint data, which makes it challenging to solve.

## Figures and Tables

**Table 1:** Comparison of DL-based approaches for LP recognition.

Ref.	Type of LP	Dataset	Technique	Accuracy	Limitations
[12]	Tunisia: Arabic characters and digits	(1) AOLP dataset: 2049 car images; (2) PKU dataset: 3977 car images; (3) Caltech dataset: 126 car images; and (4) Tunisian dataset: 740 car images	LP detection and recognition: Mask RCNN	(1) ALOP dataset: 99. 3%; (2) PKU dataset: 99.4%; (3) Caltech dataset: 98.9%; and (4) Tunisian dataset: 97.9%	(1) Working under specific lighting and weather conditions; and (2) speed of the system
[13]	Tunisia: Arabic characters and digits	GAP-LP: 9175 images	LP detection and recognition: YOLOv2	(1) GAP-LP: 97.67%; and (2) Radar dataset: 91.46%	Working under specific lighting and weather conditions
[15]	Taiwan: Latin characters and digits	AOLP dataset: 2049 car images	Modified YOLO for LP detection and recognition	LP detection: 98.22% and LP recognition: 78%	(1) Use of a single class classifier will greatly affect computation time; and (2) working under specific lighting and weather conditions.
[22]	India: Latin characters and digits	500 images	OCR	85%	(1) Videos not considered; and (2) relatively low accuracy
[23]	Pakistan: Latin characters and digits	(1) Caltech car dataset; (2) Medialab LPR dataset; and (3) own dataset	HOG and geometric features + SVM	99.3–99.8%	(1) Videos not considered; and (2) not tested on real conditions
[20]	Saudi Arabia	Own dataset: Static images extracted from 20 videos at 30 FPS using a mobile phone	LP detection: YOLOv5 and LP recognition: CNN	LP recognition: 92.8%	(1) Arabic characters are not considered; and (2) working under specific lighting and weather conditions.
[21]	Saudi Arabia	Own dataset: 1150 car images	LP detection: Faster-RCNN and LP recognition: CNN	LP detection: 92% and LP recognition: 98%	(1) Real-time videos not considered; and (2) working under specific lighting and weather conditions.
[24]	Saudi Arabia	Tested on 470 images captured in outdoor environment	Mahalanobis distance + MLP-NN classifier	94.9% for character recognition, with 1.4% rejection rate	Videos not considered
[25]	Saudi Arabia	Tested on 173 images	Character segmentation + template matching	81%	(1) Videos not considered; (2) low accuracy; and (3) lack of robustness
[26]	Saudi Arabia	350 images	Learning Vector Quantization Neural Network	94%	Videos not considered
[27]	Saudi Arabia	22 training images	Statistical features + MLP	92%	(1) Videos not considered; and (2) small dataset
[28]	Saudi Arabia	Not described	KNN	90.6%	(1) Videos not considered; (2) dataset not described; and (3) recognition of Latin characters and Western Arabic digits only
[29]	Saudi Arabia	50 images	Canny filter + OCR	92.4–96.0%	(1) Videos not considered; and (2) small dataset

**Table 8:** Evaluation of the car recognition model on a set of images.

Criterion	Value/Percentage
Number of images	100
Nonexistent car models (not included in the training dataset)	14%
Missed car detections	15%
Misclassified car models and generations	42%
Misclassified car models	38%
Correctly classified car models	38%
Correctly classified car models and generations	34%

**Table 9:** Comparison of different algorithms tested for license plate character recognition, in terms of the percentage of missed (non-detected) license plates, correctly classified characters (over detected license plates), correctly recognized license plates (all characters recognized in correct order), and processing speed in frames per second (FPS). The results were evaluated on 100 images showing cars at various distances and view angles on a Jetson Xavier AGX.

Algorithm	% Missed LPs	% Correct Characters (Single or Double)	% Correct LPs	FPS
***Model 1:*** 1-char detection (YOLOv3 320 × 320, 1 class) + image classification (MobileNetV2, 54 classes)	**18%**	86.7%	50%	4.8
***Model 2a***: 2-char detection (YOLOv4 320 × 320, 27 classes)	19%	88.3%	44%	**9.7**
***Model 2b***: 2-char detection (YOLOv4 416 × 416, 27 classes)	21%	**92.5%**	**53%**	9.5

## Data Availability

The data presented in this study are available on request from the corresponding author. The data are not publicly available due to privacy issues.
